# A Study on the Influence of Multi-Teaching Strategy Intervention Program on College Students’ Absorptive Capacity and Employability

**DOI:** 10.3389/fpsyg.2021.631958

**Published:** 2021-03-24

**Authors:** Michael Yao-Ping Peng, Lin Wang, Xiaoyao Yue, Yan Xu, Yongjun Feng

**Affiliations:** ^1^School of Economics & Management, Foshan University, Foshan, China; ^2^Research Institute of Higher Education, Yunnan University, Kunming, China; ^3^Business School, Yango University, Fuzhou, China; ^4^School of Education, Shaanxi Normal University, Xi’an, China

**Keywords:** case teaching strategy intervention program, problem-based teaching mode, absorptive capacity, student employability, higher education

## Abstract

Student employability is a key aspect of any university degree. The relationship between high student learning outcomes and high employability is a problem that needs to be addressed and improved by colleges and universities. Students with high employability can find good jobs after graduation and perform well in the workplace. Employability is associated with the success of university education, thus giving the university a good reputation. This study explores the development of employability, alongside teaching and student learning abilities to examine how these variables affect student employability. The study collected 442 responses to a questionnaire to investigate the relationship between pedagogy for employability, problem-based learning, absorptive capacity, and student employability based on a structural model. The SEM results show that pedagogy for employability and problem-based learning positively correlate to absorptive capacity; pedagogy for employability and absorptive capacity positively correlate to student employability. We then analyzed a case study teaching intervention strategy with 221 students from the school of management and discuss the differential results of all variables. Results showed that the test group was superior to the control group in each variable performance, indicating that the intervention strategy proved effective. Based on these findings, this study proposes suggestions for future research.

## Introduction

Higher education is crucial to national economic progress as it enables human resources to grow (Choi and Rhee, 2014). Based on the statistics of the Ministry of Education in Taiwan, there were 152 higher educational institutes (HEIs) in the country by 2019, showing that the quantity of HEIs has reached saturation and the educational system has gradually changed (Teow, 1984; Taylor et al., 2013). Although higher education reform policy has afforded more education opportunities for students, many problems, such as inferior teaching quality and competitiveness have also risen to become latent problems that affect the development of higher education in Taiwan (Shin and Harman, 2009; Marginson, 2011). Recent studies on HEIs have demonstrated that student learning outcomes (LOs) can be greatly enhanced via the improvement of teaching quality and the optimization of resources and equipment (Pike et al., 2011, 2012; Maringe and Sing, 2014).

Few studies have done a survey, from the perspective of the job market and talent flow, on the connection between student learning and student employment. Students in tertiary institutions have developed many skills and abilities, indicating that there is high engagement in learning. Vygotsky (1994) argued that students are dedicated to developing their elaborative faculty during college learning. When proper tasks are created in the learning environment or classroom to stimulate students’ intelligence, students change their inherent thought processes and they are inspired to reach their learning potential. In other words, classroom teaching should shape a substantial real-world environment and guide students in their ability to recognize the capabilities required for problems and tasks in the employment environment, including problem-solving and reflective capabilities (Vygotsky, 1998). For students to return to the job market, colleges need to emphasize student employment instead of enhancing the efficiency of student learning. De Vos et al. (2011) outline that employability involves knowledge acquisition, skills obtainment, and other characteristics that are required to satisfy the needs of employers and achieve professional potential. The stimulus-organism-response (S-O-R) model was developed in response to the idea that humans are organisms that generate psychological elements such as feelings, moods, emotions, or attitudes in response to stimuli (Khan et al., 2017; Kim and Park, 2019; Zhai et al., 2020). To explore the learning responses of students to the teaching pattern explored here, and to ensure that student employability serves as the main response in this study, we used the stimulus-organism-response (S-O-R) model and connected it with student employability.

As for employment requirements, mastery of knowledge and expertise at university level ensure that students can use their skills in a diverse workplace (Bridgstock, 2009; Crossman and Clarke, 2010). Encouragement and guidance from teachers can contribute to improving student employability and enhances their motivation, input, and efficiency when learning (Robinson et al., 2008). Taking Vygotsky’s (1994, 1998) proposition on the student learning process, the first thing they need is to develop self-awareness (i.e., the perception and cognition of the employment market), they then need to further comprehend the importance of social interaction, and familiarize themselves with the language and process of communication. These are implied in the process of developing the employability of students and internalizing external knowledge, which comes largely from interpersonal communication and interaction, among which the role of the teacher is exceedingly important (Vygotsky, 1978). Instructors facilitate a positive learning attitude and develop future employability through the integration of well-defined employability activities in class. Building upon this concept from Vygotsky, this study adopted two teaching patterns that are based on employment-oriented teaching activities and problem-based learning (PBL), which are viewed as the main sources of stimulus in the S-O-R model. In particular, problem-based learning (PBL) motivates students to invest more under different learning circumstances, from problem solving and relevant information searching, to strategic solution planning, thereby enhancing their learning effectiveness (Chang et al., 2012) and serving as the foundation for student employability in the future. Based on the above arguments, this study aims to explore the influences of employment-oriented teaching activities and problem-based learning on student employability.

According to Zimmerman (2002), self-regulated learning explains individual differences, from stress on the learners’ ability to prepare for differences and their dynamic comprehension of learning, emphasizing a learners’ ability to self-regulate. Therefore, the intrinsic motivation of learners is affected by how they are introduced to their own psychological resources, and how they account for environmental information. Bruner (1978) discusses intrinsic motivation and intellectual development, arguing that intrinsic motivation is a genuine motivation for learning. Students with strong intrinsic motivation can fully master basic and initial knowledge and skills, which provide the psychological foundations for establishing deeper knowledge and skills. Fabrizio (2009) underlined that students need a transformation approach of knowledge to improve learning, digestion, transfer, and utilization of knowledge, to illustrate the efficiency of knowledge. Thus, in this study, absorptive ability is a significant factor of the S-O-R model, serving as the mediator in the model.

## Literature Review and Hypotheses Development

### Student Employability (SE)

A number of scholars are currently dedicated to employability-related research. Major technological, social, and economic vicissitudes have emerged in recent decades (Abbas et al., 2015) that have changed and improved the ideas and operation of industrial organizations (Abbas and Sağsan, 2019) and HEIs worldwide (Vermeulen et al., 2018). The development of human capital is of high importance and informs the standards of dynamic HEIs as it is conducive to economic growth (Ahmed et al., 2015; Baek and Cho, 2018).

By studying research situations and method designs, as well as combining theoretical analysis and practical analysis, scholars have explored the significance of employability and the causal relationship between employability and other elements (Hennemann and Liefner, 2010; Avramenko, 2012; Baek and Cho, 2018). According to Heijde and Van Der Heijden (2006), employability refers to the best use of individual capabilities (Blázquez et al., 2018), subsequent learning and creation of basic technical proficiencies for overall task achievement and adaptation to internal and external vicissitudes in the employment market (Fugate et al., 2004; Cuyper et al., 2008; Vermeulen et al., 2018; Malik et al., 2019). Thus, it is increasingly necessary to have critical and reflective thinking, the ability to settle matters, autonomous management and learning, as well as other related abilities, across all disciplines (Makkonen and Olkkonen, 2017). Previous studies have shown that fundamental education affects not only employability, but also other factors, including individual conditions and interpersonal connections, as well as factors which can not be obtained from higher education (Ahmed et al., 2015; Cacciolatti et al., 2017; Blázquez et al., 2018).

### Pedagogy for Employability (PE)

In the field of student learning issues, learning outcomes are persistently explored by scholars. Through the observation of behavior, learning outcomes measure large knowledge acquisition, construction updating, comprehension deepening, and discussion, and how learners attempt to accomplish a specific task that needs elementary knowledge (Oleson and Hora, 2014; Ahmed et al., 2015; Al-Jubari et al., 2019). Thus, passive and teacher-oriented teaching methods need to be eliminated and active and learner-centered activity design must be adopted by teachers in order to inspire the learning orientation and engagement of students. Teachers need to be dedicated to encouraging students who are involved in deeper comprehension. Then students can adopt real-world examples in different contexts (Tagg, 2003; Al-Jubari et al., 2019). Universities should provide better networking and connections with industry and ensure that students learn the knowledge and skills required for employment (Corbett, 2005; Unruh et al., 2008; Ahmed et al., 2015; Blázquez et al., 2018). Therefore, teachers ought to motivate students to acquire the orientation and input process based on active, learner-oriented activity designs, and encourage students in deepening comprehension and their understanding of the meaning of subjects, so that they can utilize these skills in real-world paradigm in various surroundings (Tagg, 2003; Baek and Cho, 2018).

Based upon the different purposes of student cultivation, teaching materials should also be adjusted. Bruner (1966) proposed that the structure of subject matter and the structure of a subject improve knowledge and experience in terms of the ‘quality’ of textbooks, focussing on cultivating students’ attitude toward active learning and exploration, analogy, and problem-solving, as well as acquiring a sense of excitement through discovery. In ‘Pedagogy for Employability’ Yorke (2006) discusses how governments all over the world have accepted the close connections between higher education and the national economy, which also implies the significance of human capital perspectives (Becker, 1975; Baek and Cho, 2018; Blázquez et al., 2018). The economic interests of creativity, entrepreneurship, and entrepreneurship in the workplace (Al-Jubari et al., 2019) is also underlined by Reich (2002) and Reed (2002), indicating that both the quality of the factors and the surroundings in which skills are developed impact economic advancement. Higher education needs to have a more entrepreneurial spirit, to position student expectations and ensure that discipline, employment skills, understanding, and quality of education can be accelerated. Current concerns about student employment issues can thus be addressed by employment-oriented curriculum design and learning activities, illustrating the connection between pedagogy for employability and professional disciplines (Ahmed et al., 2015; Baek and Cho, 2018), indicating that design is not a substitute.

Yorke and Knight (2004) concentrate on embedding teaching activities and employability in the design of curriculum, making teachers equipped with the ability to adjust the curriculum structure and enhance it to offer better and more effective practices in pedagogy for employability (Baek and Cho, 2018). Nevertheless, few studies mention an explicit measurement of employment-oriented teaching activities. Yorke (2006) proposes several designs for employment-oriented teaching activities. The principles of this study concentrate on pedagogy for employability that establish a measurable scale for analyzing the case-study. These include annotating a bibliography instead of writing ‘yet another essay’; making critical commentaries or reviews, maybe in the style of a particular type of publication; generalizing complicated material in the form of a brief or an outline; establishing criteria for performance judgment; figuring out problems in a group, involving attention in the group dynamics of teamwork; in-tray exercises, perhaps with a time limit; doing a case presentation, and being prepared to plead for it; role-playing; and surveying public perceptions, for example by collecting oral history data or consumer preferences.

Teachers need to aid students in the enhancement of their employment conditions through internal and external inducements (Bogler et al., 2013). Since students’ external learning output and academic achievements are reflected by general and professional ability, a high degree of learning satisfaction is needed from students to meet these conditions (Likisa, 2018). When students are content with their learning status, they are capable of fulfilling better academic achievements or learning outcomes (Bogler et al., 2013; Baek and Cho, 2018; Al-Jubari et al., 2019), a fundamental basis for the development of employability. Specific course design and the implementation of teaching content, methods and attitudes, and the interaction between teachers and students in the program are available for teachers in class (Corbett, 2005; Hmelo-Silver et al., 2007) and depending on their application in an educational environment, studying pedagogy for employability and practical experience acquisition contributes to the exploration of the relationship between class activities and employability. Based on these results, schools and teachers are aware of the most effective curriculum planning and activities.

Based on the above discussion, this study proposes the following hypotheses:

H1: Pedagogy for Employability will positively correlate to student’s absorptive capacity.
H2: Pedagogy for Employability will positively correlate to student’s employability.

### Problem-Based Teaching Mode (PBTM)

As a type of learning model, PBTM has received more attention in recent years (Savery, 2006; Koh et al., 2008; Schmidt et al., 2011). PBTM emphasizes that students are the main focus of teaching, and also divides the learning process into five stages: ‘question raising –hypothesis establishment – information collection – argument over hypotheses – summary.’ It also serves as a learning context in which students are placed in a complicated but meaningful problem situation so that they can learn to solve through teamwork and undertake knowledge acquisition via this process. They, therefore, develop and achieve deeper problem-solving and self-learning abilities (McGrath et al., 2006).

Problem-based Teaching Mode had been adopted by teachers in the past to enhance students’ exploration of knowledge (Chang et al., 2012). In the present study, PBTM has the following characteristics: (1) student-centered learning patterns; (2) easier implementation in student groups that are teacher-led and small-scale; (3) teachers acting as assistants or guides; (4) problem discovery is crucial to knowledge acquisition and problem settlement; and (5) self-guided learning which is more accessible to obtainment is not limited to essential information and ability to settle problems when they occur (Hmelo-Silver et al., 2007; Chang et al., 2012). That is, PBTM arises from the learning needs of students. Active engagement, knowledge, and practical skills can be enhanced by teachers with strategies of inquiry, cooperation, and reflection.

In previous studies, the learning opportunities available to individuals have a positive effect on better performance and improve self-efficacy (Zhao et al., 2005; Al-Jubari et al., 2019). In addition, to show the impact of self-efficacy, students engage in the progress of long-term proper learning experiences that would have a positive effect on tasks and objects under the personal ability (skills) and action (challenge) of subjective assessment (Likisa, 2018). When students face activities that need more knowledge and other challenges, there is more individual investment from students to conquer the challenges and gain a proper learning experience. Thus, not only the internal incentives but also the design of diversified learning activities should motivate students to probe into learning implications when acquiring knowledge, set their long-term learning goals (Delle Fave and Massimini, 2005), and predict their future career (Bassi et al., 2007).

In the learning environment, students with higher self-efficacy could facilitate their academic interests, emotional management, learning incentive, cognitive competence, and performance growth (Bandura et al., 1996; Bandura, 1997). There also tends to be a strong mediating effect on individual achievements and self-practice upon follow-up (Lent and Brown, 2006). Dunlap (2005) found that PBTM contributes to the acquisition of expertise and skills for students that are necessary when they work. Nevertheless, improving learning achievements without the prerequisite of self-efficacy is difficult. Thus, when it comes to the teaching strategy of PBTM, not only the establishment of short-term goals but also the establishment of long-term goals needs emphasis, along with feedback that concerns students’ learning performance, so that their self-efficacy is improved. Hypothesis 3 of this study thus proposes that:

H3: Problem-based teaching mode will positively correlate to student’s absorptive capacity.

In terms of student employability, a problem-based teaching model is conducive to enhancing the interest of students in acquiring and utilizing their professional skills, further improving the capability of students (Spronken-Smith, 2005; Martin et al., 2008). Students are able to undertake better learning and critical thinking in face of practical problems, such as critical analysis, problem settlement, and reflection. Duncan and Al-Nakeeb (2006) have confirmed that students involved in the problem-based teaching method change their learning incentives, attitudes, and behaviors, with improved critical thinking, learning autonomy, and capabilities that are relevant to employment. Thus, hypothesis 4 of this study proposes:

H4: Problem-based teaching mode will positively correlate to student’s employability.

### Absorptive Capacity (AC)

Mariano and Pilar (2005) defined AC as individual abilities to discover new knowledge, acquire valuable knowledge, and utilize this knowledge to achieve major purposes. Moreover, the enhancement of students’ capacities and skills will determine how they utilize, combine, and even fundamentally develop core capabilities. Absorptive knowledge plays a crucial part in the process of knowledge conversion, and students need to internalize new external knowledge through the socialization process (Yeoh, 2009). Being embedded in a relationship of mutual benefit and collaboration and trust, students and teachers will enhance the efficiency of communication and knowledge transfer as the interaction between them increases. Jiang et al. (2008) argue that if students have a good absorptive capability, they can improve their capability to utilize knowledge learned in class, absorb external knowledge more efficiently, and deepen understanding of new external knowledge, thus enhancing their general and professional capabilities. In other words, if students are not equipped with absorptive capability, they are unable to completely absorb and employ tacit or explicit knowledge transferred from teachers (Chen, 2003). Students with sufficient absorptive capability conduct open communication and exchange of knowledge through common interest and language and reserve valuable knowledge (Wenger, 1998; Cadiz et al., 2009), which improves employability. Nor et al. (2012) indicated that students who are equipped with absorptive capability perform well in terms of academic attainment and prior knowledge and that they can transfer and employ knowledge in an efficient manner, thus improving their academic performance and employability in the future.

Employability comprises knowledge, technology, and variety (Hennemann and Liefner, 2010; Baek and Cho, 2018). In the context of higher education, students can lack sufficient knowledge and ability to absorb (Blázquez et al., 2018). In other words, adequate AC can enable students to exchange and share knowledge relevant to the connotations of knowledge via mutual interest and language, leading to the acquisition of valuable knowledge (Wenger, 1998; Cadiz et al., 2009; Abbas and Sağsan, 2019). AC has positive effects on employability. Nor et al. (2012) also found that it is beneficial for students to learn AC because it equips them with prerequisite knowledge and academic achievement. Thus, knowledge transfer and application can be conducted effectively, and their academic achievement and employability development can be improved. Based on this description, the following hypothesis this study deduced the following hypothesis:

H5: Students’ absorptive capacity will positively correlate to employability.

### Multi-Teaching Strategy Intervention Program

Different teaching patterns affect students’ understanding of a course and their wider knowledge of the subject, affecting learning engagement, and leading to different learning motivations. In previous research, multiple teaching strategies have been proposed, which include case studies, traditional teaching, modern teaching, and other alternative methods, etc. (Noblitt et al., 2010; Nurutdinova et al., 2016). Noblitt et al. (2010) compared the differences between case studies and traditional teaching methods and stated that case studies are a more effective approach for teaching scientific oral communication skills. However, the cultivation of student employability discussed in this research differs from previous research. To verify whether employment knowledge has different effects on research variables via different teaching strategies, this study proposes a multi-teaching strategy (case and traditional teaching). The case teaching strategy has achieved good results in different fields (Dochy et al., 2003; Hoag et al., 2005; Yadav and Beckerman, 2009). The reason why this study chose this case teaching strategy as the intervention for testing the differences between the experimental group and the control group is that case teaching strategy and employability are closely connected. This makes it necessary for students to take action in the simulated scene, and to understand the management and marketing mode of enterprises, to achieve the integration of theory and practice and then feedback on improved employability (Yadav et al., 2011). Thus, this study explores the impact of employment-oriented teaching activities, problem-oriented learning on absorptive capacity and employability using a case study teaching strategy. According to the above arguments, the hypothesis for this study is as follows:

H6: Students who receive teaching by “case teaching strategy” in the experimental group are more satisfied than students who receive traditional teaching in the control group.

**FIGURE 1:**
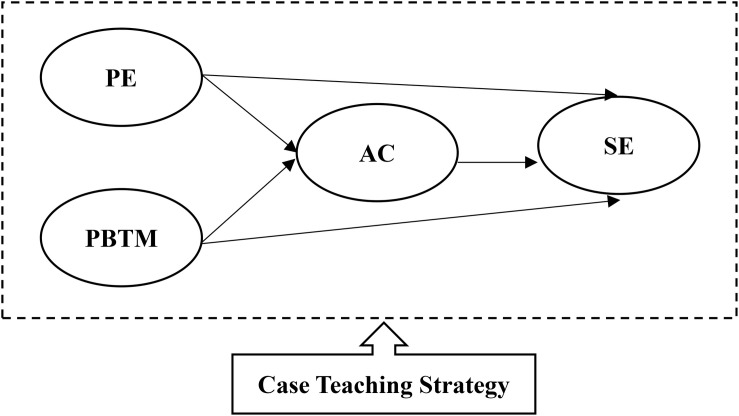
Research Framework.

Based on the above research motivation and the research significance, as outlined in the plan, we aimed to verify the following research framework, shown in Figure 1:

## Methodology

### Sampling

This study uses a quasi-experimental design. To reduce errors in the quasi-experimental design and increase the representativeness of samples, the study divided students from the same course in the Business School into two groups. One class adopted a case teaching strategy (Experimental group, *N* = 120) and the other class used traditional teaching strategies (Control group, *N* = 101) to explore the differences in teaching outcomes between the two groups. Since this study is a quasi-experimental design, we did not adopt random assignment and depended on the post-mortem statistical control. This is one of the important limitations of this study. Nonetheless, this study was conducted based on normal teaching in the school, and the experimental treatment received by students in the control group was comparable to that of the experimental group, which also increases the credibility of the research results.

### Procedure

The study began in September 2019 and ended in January 2020. We contacted the educational center and the designed case teaching study was applied to Marketing courses. Apart from the mid-term and final exam weeks, one to two hours of case teaching was undertaken each week, supplemented by case discussions. This study aims to explore differences in case teaching strategy between the experimental and control groups in terms of case structure and variables. Therefore, students were grouped at the beginning of the first week of the semester, and each group included 4 to 5 people. Each group was assigned a group leader, and each member of the group took turns making comments and forming opinions about the discussion during the case discussion of each week.

In terms of the design of courses, the experimental group was not like the control group. The teacher provided case questions on employment, which were discussed by students in the experimental group. This trained students in teamwork by encouraging them to discuss practical issues assigned by the teacher. Based on the case teaching strategy, the teacher designed courses combining problem-oriented learning and employability-oriented teaching activities. In addition to reporting the results of the case discussion, students also needed to answer questions raised by the teacher and students of the other group, until there were no questions raised by students. The control group used the traditional teaching mode, where the teacher explains the course contents from the first week to the last week, to increase the difference between the experimental and control groups.

### Variable Measure

Student employability can be referred to as a socio-psychological construct that includes subjective and objective aspects (De Vos et al., 2011), thus the evaluation of SE is also classified into subjective and objective aspects. Regardless of the scales developed by scholars (Pan and Lee, 2011) or government organizations (Department of Education, Science and Training [DEST], 2006), which all have certain reliability, validity, and degrees of generality, it is difficult for students to objectively evaluate their learning process. Subjective self-evaluation was adopted as the main evaluation method in this study. This study includes the general ability for work (GAW) (8 items), the professional ability for work (PAW) (4 items), the attitude at work (AW) (3 items), and career planning and confidence (CPC) (3 items) measures, as proposed by Pan and Lee (2011). For PBTM, the scales developed by Chang et al. (2012) were adopted, including knowledge-sharing (KS) (3 items) and problem-solving (PS) (3 items). In the POE, this study adopted items proposed by Pegg et al. (2012) that were converted into a measurable scale to measure the perceived level of pedagogy for employability. Following Lee (2007) and Cadiz et al. (2009), absorptive capacity was measured in terms of assessment (3 items), assimilation (3 items), and application (3 items). All items have been measured on a five-point Likert scale (1 = totally disagree; 5 = totally agree).

## Analysis and Results

### Reliability and Validity

All scales used in this study were found to be reliable, with Cronbach’s α ranging from.83 to.96. Table 1 shows the reliability of each variable. To verify validity, this study adopted confirmatory factor analysis (CFA) to verify the construct validity (both convergent and discriminant) via AMOS 23.0. According to Hair et al. (2006) suggestions, CFA results show standardized factor loading of higher than 0.5; average variance extracted (AVE) ranges between 0.63 ∼ 0.88, and composite reliability (CR) ranges between 0.83 ∼ 0.95. All three conditions for convergent validity were met, and correlation coefficients were all lower than the square root of the AVE within the same dimension, suggesting that each dimension in this study had good discriminant validity.

**TABLE 1:** Measurement.

	1	2	3	4	5	6	7	8	9	10
1.GAW	***0.82***									
2.PAW	0.790**	***0.80***								
3.AW	0.789**	0.762**	***0.79***							
4.CPC	0.786**	0.762**	0.757**	***0.83***						
5.PE	0.687**	0.650**	0.678**	0.700**	***0.81***					
6. Assessment	0.584**	0.563**	0.468**	0.473**	0.557**	***0.94***				
7. Assimilation	0.500**	0.525**	0.425**	0.428**	0.543**	0.854**	***0.88***			
8. Application	0.564**	0.523**	0.459**	0.456**	0.627**	0.868**	0.869**	***0.89***		
9. KS	0.533**	0.522**	0.489**	0.485**	0.553**	0.742**	0.717**	0.737**	***0.91***	
10.PS	0.479**	0.482**	0.414**	0.395**	0.559**	0.775**	0.818**	0.819**	0.819**	***0.88***
Means	5.67	5.58	5.65	5.46	5.51	5.61	5.86	5.62	5.32	5.85
SD	1.22	1.34	1.33	1.54	1.35	1.68	1.80	1.73	1.85	1.76
Crobach’s α	0.87	0.88	0.82	0.86	0.95	0.93	0.91	0.92	0.93	0.91
AVE	0.68	0.64	0.63	0.68	0.65	0.88	0.78	0.80	0.82	0.77
CR	0.88	0.88	0.83	0.87	0.95	0.93	0.92	0.92	0.93	0.91

Bold values: Square root of AVE for each latent construct is given in diagonals; **p < 0.05.

### Inner Model Analysis

Henseler et al. (2014) indicated the SRMR is a goodness of fit measure that can be applied to avoid model misspecification. NFI values above 0.9 usually represent acceptable model fit. In this study, the SRMR value was 0.055 (< 0.08) and the NFI was 0.912 (> 0.90), and the *d*_*ULS*_ < bootstrapped HI 95% of *d*_*ULS*_ and *d*_*G*_ < bootstrapped HI 95% of *d*_*G*_, indicating that the data fits the model well. To assess the structural model, Hair et al. (2017) suggested focusing on the *R*^2^, β, and the t-values via a bootstrapping procedure with a resample of 5,000. They also indicated that researchers should also show predictive relevance (*Q*^2^) as well as the effect sizes (*f*^2^). The VIF values were less than 5 (Hair et al., 2017), ranging from 1.314 to 1.773.

**FIGURE 2:**
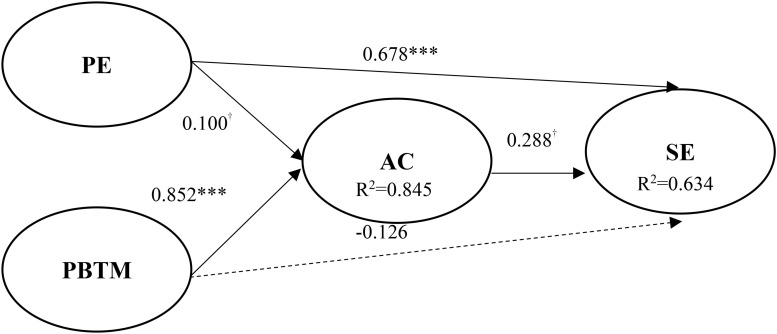
Path analysis. ***p < 0.001; ^†^employability.

Findings are shown in Figure 2. The path coefficients of pedagogy for employability to students’ absorptive capacity and employability were 0.100 (*p* < 0.1) and 0.678 (*p* < 0.001), so H1 and H2 were supported. Moreover, the problem-based teaching model (β = 0.852, *p* < 0.001) was positively and significantly related to absorptive capacity, supporting H3. This implies that teachers with more problem-based teaching activities facilitate the improvement of students’ absorptive capacity. Similarly, students’ absorptive capacity was positively and significantly related to student employability, supporting H5. However, the path coefficient of the problem-based teaching model (β = −0.126, *p* > 0.1) to student employability was negative and insignificant, which did not support H4. The Stone-Geisser *Q*^2^-values obtained through the blindfolding procedures for student absorptive capacity (*Q*^2^ = 0.337) and student employability (*Q*^2^ = 0.384) were larger than zero, supporting the conclusion that the model has predictive relevance (Hair et al., 2017).

### The Intervention Differences of Teaching Programs

In this study, the differences between the case teaching strategy and the traditional teaching strategy, between pretest (M1) and posttest (M2) were tested by the t test of paired samples, in order to understand whether the teaching was effective. As shown in Table 2, except for the PS (*t* = −1.43; *p* > 0.10) in the control group, there are significant differences in other variables. This means that both case teaching strategies and traditional teaching strategies can improve students’ learning outcomes.

**TABLE 2:** A Study of the independence value between the control groups and experimental groups.

	Experimental group					Control group				
	M1		M2		M1-M2	M1		M2		M1-M2
	M	SD	M	SD	t	M	SD	M	SD	t
SE scale	5.59	1.54	6.95	1.56	−4.60***	5.53	1.15	6.45	1.37	−4.40***
GAW	5.76	1.51	7.00	1.54	−4.29***	5.57	1.05	6.35	1.38	−3.68**
PAW	5.51	1.58	6.98	1.56	−4.86***	5.59	1.27	6.35	1.38	−4.25***
AW	5.66	1.65	6.94	1.61	−4.10***	5.51	1.23	6.43	1.53	−3.95***
CPC	5.45	1.69	6.87	1.62	−4.44***	5.47	1.49	6.44	1.55	−3.92***
PE scale	5.66	1.67	6.92	1.62	−3.77***	5.43	1.51	6.46	1.34	−5.11***
AC scale	5.81	1.75	7.04	1.68	−3.60**	5.78	1.28	6.38	1.36	−2.77**
Assessment	5.80	1.76	7.07	1.64	−3.88***	5.68	1.35	6.39	1.35	−3.03**
Assimilation	5.89	1.76	7.03	1.73	−3.21**	6.01	1.46	6.44	1.43	−1.81^†^
Application	5.75	1.79	7.02	1.73	−3.61***	5.64	1.44	6.31	1.40	−2.84**
PBTM scale	5.74	1.69	7.10	1.61	−4.03***	5.80	1.37	6.29	1.34	−2.13*
KS	5.61	1.69	7.07	1.61	−4.37***	5.49	1.56	6.13	1.43	−2.48*
PS	5.87	1.77	7.12	1.65	−3.57**	6.10	1.37	6.45	1.39	−1.43

Note: * if *p* < 0.05; ** if *P* < 0.01; ***p < 0.001; ^†^employability.

This study tested the differences between the experimental group and the control group by performing a t test of independent samples in terms of scale scores in the pre-test and post-test (see Table 3). In the t test of pretest independent samples, it was found that the differences between the experimental group and the control group in terms of variables were statistically insignificant. This indicates that there was no difference between the two, and also confirms the feasibility of making structural equation modeling analysis by the overall pre-test samples. In terms of post-test scores, it is known from the measurement variables that GAW (*t* = 2.19; *p* < 0.05), assessment (*t* = 2.13; *p* < 0.05), assimilation (*t* = 1.83; *p* < 0.10), application (*t* = 2.21; *p* < 0.05), KS (*t* = 3.07; *p* < 0.01) and PS (*t* = 2.17; *p* < 0.05) are significantly different. From the perspective of constructs, the post-test scores of SE (*t* = 1.69; *p* < 0.10), AC (*t* = 2.13; *p* < 0.05), and the PBTM (*t* = 2.69; *p* < 0.01) of the experimental group are superior to that of the control group. There is no significant difference in employment-oriented teaching activities. Therefore, H6 is partially supported.

**TABLE 3:** A study of the independence value between experimental and control groups.

	M1					M2				
	Experimental		Control			Experimental		Control		
	M	SD	M	SD	t	M	SD	M	SD	t
SE scale	5.59	1.54	5.53	1.15	0.22	6.95	1.56	6.45	1.37	1.69^†^
GAW	5.76	1.51	5.57	1.05	0.73	7.00	1.54	6.35	1.38	2.19*
PAW	5.51	1.58	5.59	1.27	−0.28	6.98	1.56	6.35	1.38	1.36
AW	5.66	1.65	5.51	1.23	0.52	6.94	1.61	6.43	1.53	1.60
CPC	5.45	1.69	5.47	1.49	−0.07	6.87	1.62	6.44	1.55	1.34
PE scale	5.66	1.67	5.43	1.51	0.83	6.92	1.62	6.46	1.34	1.54
AC scale	5.81	1.75	5.78	1.28	0.12	7.04	1.68	6.38	1.36	2.13*
Assessment	5.80	1.76	5.68	1.35	0.38	7.07	1.64	6.39	1.35	2.27*
Assimilation	5.89	1.76	6.01	1.46	0.38	7.03	1.73	6.44	1.43	1.83^†^
Application	5.75	1.79	5.64	1.44	0.35	7.02	1.73	6.31	1.40	2.21*
PBTM scale	5.74	1.69	5.80	1.37	−0.18	7.10	1.61	6.29	1.34	269**
KS	5.61	1.69	5.49	1.56	0.35	7.07	1.61	6.13	1.43	3.07**
PS	5.87	1.77	6.10	1.37	−0.72	7.12	1.65	6.45	1.39	2.17*

Note: * if *p* < 0.05; ** if *P* < 0.01; ***p < 0.001.

## Discussion and Implications

This study aims to explore the curriculum and literature relevant to teaching to provide a better understanding of the concept of student employability, in terms of its nature and meaning. Previous studies on employability have been reviewed, with a focus upon enterprise. To date, few studies concentrate on the employment ability of higher education institutions via the particular employment-oriented teaching curriculum from the teachers. Most previous studies on the S-O-R model have focused on the importance of external environmental stimuli (Khan et al., 2017; Kim and Park, 2019; Zhai et al., 2020), but few studies have analyzed the role of teaching factors in fostering employability. This study, therefore, aimed to fill these gaps by conducting a survey of this area and exploring the application of the S-O-R model. In addition to verifying the research framework established by the S-O-R model, we obtained relevant findings with regard to the academic achievement of students and discussed the course curriculum in relation to teaching and student learning, and how these form student employability. A quasi-experimental design was also adopted to verify the relationship between the overall model frameworks, thereby proposing richer insights and implications for the theory of employability.

This study found that PE has a positive effect on the absorptive capacity and employability of students. This result is similar to those of Corbett (2005); Lent and Brown (2006), and Hmelo-Silver et al. (2007). Our study built upon this past research, by discussing the hierarchical characteristics of student capability cultivation, namely, absorptive capacity and employability. While these are capabilities needed by students when they enter the workplace after graduation (Vermeulen et al., 2018; Abbas and Sağsan, 2019), the results of the present study indicate that even with a higher level of improvement to capabilities, absorptive capacity may not lead to superior employability.

This study also explored employment-oriented teaching activities adopted by teachers in the classroom environment to stimulate students’ cognition of employment and joy in problem discovery. As Cadiz et al. (2009), Blázquez et al. (2018) and Abbas and Sağsan(2019) emphasized in their studies, developing students’ capability to internalize knowledge via knowledge acquisition, transformation, application, and repeatedly promoting the effect of knowledge spiral, further enhances the capabilities needed for the employment market. Thus, to cultivate high employability for students, teachers must take student learning processes and competence evolution into consideration in curriculum design. Bruner (1966) suggests that pedagogy design for employability should induce students to see the fun of learning employment knowledge and guide them to form learning mechanism capabilities of inner knowledge-circulation. Although an overall comprehension of the ties between education and industries exists, there is a lack of explicit understanding of how to implement a flipped classroom model and conditions for the enhancement of teachers’ employment-oriented teaching activities (POE). This study advises teachers that they need to understand the conditions and standards of industry and embed these into the design of curricula and teaching activities to enhance student employability and facilitate flipped learning.

Another interesting finding of the present study is that problem-oriented teaching activities may have a negative effect rather than a significant effect on student employability. Nevertheless, we observed positive effects on the absorptive capacity of students and a significant mediating effect between the problem-centered teaching model and student employability. These results echo the self-regulated learning theory of Zimmerman (2002) and the model of employability established by Hennemann and Liefner (2010), which not only show that student employability is necessarily tied to the development path simulating the teaching and learning context for the enhancement of students’ skills and capabilities, but also have a deeper formulation of their employability. These research findings show that the path coefficient of problem-oriented teaching activities→absorptive capacity is higher than that of employment-oriented teaching activities→absorptive capacity, which means that problem-oriented teaching activities are more conducive to enhancing students’ absorptive capacity. Problem-oriented teaching activities should focus on the students’ ability to efficiently utilize their knowledge, address problems, and acquire new knowledge. Moreover, problem-discovery and problem-solving in problem-oriented activities induce students to reflect and explore during knowledge learning, which is more in line with the process of knowledge acquisition, transformation, and internalization of absorptive capacity. Even though the overall effect of problem-oriented teaching activities→student employability is lower than that of employment-oriented teaching activities→student employability, problem-oriented teaching activities still play a driving role in the process of inspiring students to take the initiative to learn curriculum knowledge to develop relevant capabilities, such as continuous learning and the development process as advocated in the S-O-R model (Animesh et al., 2011; Ha and Im, 2012; Zhai et al., 2020). The application of quasi-experimental designs in exploring the employability of students is a methodological breakthrough in literature. Research results show that there is no significant difference between the experimental group and the control group in the pre-test, but multiple variables were found to be significantly different in the post-test. This is the result of the intervention by experimental design, and also confirms the feasibility of the case teaching strategy in the employability model. This result is consistent with another study by Yadav et al. (2010). The case teaching model can enhance students’ critical thinking, problem-solving skills, advanced thinking skills, and learning motivation. Applying this to the design of the experimental and control groups in the employability model will provide valuable implications for employability theory.

### Teaching Implications

This study found that pedagogy for employability positively affects the absorptive capacity and employability of students, which suggests that teachers must integrate critical elements in future employment in the design of course content and teaching models, and avoid putting excessive emphasis on the fixed recitation of the course content. At present, many universities are aiming to enhance the link between industry and classroom, but ways of developing teachers’ capacity for pedagogy design by adding materials relevant to employability into the teaching system remain unclear.

This study has suggested that understanding the conditions and standards of curriculum and teaching activities is necessary for teachers to enrich students with skills crucial to employability, thus leading to the successful creation of flipped learning. Problem-oriented teaching activities center on students who can effectively address problems with their own knowledge and gain new knowledge during the process. Students need problem-solving skills to participate in the teaching situation and to make use of their acquired knowledge and skills in future work. Hence, in this study teachers were advised to pay more attention to teaching curriculum design and teaching activities, so that students can consider the elements again when they solve problems, and to further afford help to them with regard to the assimilation, conversion, absorption, and understanding of knowledge applications.

To facilitate the development of a positive attitude toward academic learning in students, the present study suggests that teachers’ curriculum content design and teaching models are necessarily combined with practice, diversified activities, and case analysis, thus leading to participation enhancement and knowledge improvement in students. Additional attention to students’ motivation to learn should be paid by teachers. These findings indicate that students engaged in case discussion and result sharing are more inclined to challenge themselves, make active investments and establish stronger self-confidence. In addition, combining the activities of teaching and student inquiry motivates students to engage in active learning and develop their employability.

This study repeatedly verifies the effectiveness of case teaching strategies, employment-oriented teaching models, and problem-oriented learning on student employability, indicating the importance of teaching model design. Based on these results, this study suggests that teachers should continue to encourage students to reflect on the learning process, and enrich teaching with reference to each student’s case discussion and comments. In the case teaching strategy, students reflected on different aspects of their learning, including discussion and the application of professional knowledge and cross-domain knowledge. Thus, this case teaching strategy not only helps students to acquire professional knowledge, its process of team discussion and reflection is also an important method that enables teachers to understand student learning and identify students’ learning difficulties.

### Research Limitations and Suggestion for Further Studies

Even though these research results contribute to literature on student learning, some limitations still exist and suggest further research directions. First, self-regulated learning theory has obtained considerable status in the psychological field, but only a few studies have considered the relationship between absorptive capacity and the employability of undergraduate students in higher education. Although the teaching methods of this study (Problem-based teaching mode and Pedagogy for Employability) were constructed with reference to self-regulated learning theory, and important learning theories can be derived from the research results, other motivation theories, such as Self-determination theory, Social-cognitive theory, and achievement motivation theory, can still be used to trigger learning in undergraduate students. Thus, future research should utilize different theoretical models to identify the relevant teaching strategies that influence students’ capacity and employability. Second, this study required students to self-report on teachers’ teaching methods, mainly because actual data is confidential and not easily obtained. However, errors may exist in the students’ self-statement of their psychological status. The link between teaching methods and employability may be better understood if students’ actual psychological status were assessed, with due consideration for research ethics. Future researchers should include interviews and observations about students’ learning status to support the research results and enable a more comprehensive judgment. Third, due to restrictions of time and space, two classes were sampled in this study, with 442 valid questionnaires in total. The research objects were divided into experimental and control groups of Business School students. Future research could explore and compare other groups on different courses, expand the quantity of samples, and improve the representativeness of the research to provide additional insights relevant to student employability.

## Data Availability Statement

The raw data supporting the conclusions of this article will be made available by the authors, without undue reservation.

## Ethics Statement

The studies involving human participants were reviewed and approved by University of Taipei. The patients/participants provided their written informed consent to participate in this study. Written informed consent was not obtained from the individual(s) for the publication of any potentially identifiable images or data included in this article.

## Author Contributions

MP, YF, and LW contributed to the ideas of educational research, collection of data, and empirical analysis. MP, LW, YF, YX, and XY contributed to the data analysis, design of research methods, and tables. MP, YX, and XY participated in developing a research design, writing, and interpreting the analysis. All authors contributed to the literature review and conclusions. This study is a joint work of the all authors.

## Conflict of Interest

The authors declare that the research was conducted in the absence of any commercial or financial relationships that could be construed as a potential conflict of interest.
